# American Institute of Homœopathy

**Published:** 1893-11

**Authors:** Wm. E. Leonard

**Affiliations:** Secretary


### AMERICAN INSTITUTE OF HOMŒOPATHY,

### Bureau of Materia Medica and Therapeutics, 1892–3–4.

The following circular has been issued by this Bureau. Any practitioner having definite views upon this vital topic is requested to join in the symposium and correspond with the Secretary:

Dear Doctor:—In organizing the work of this Bureau for the year 1894 (the session of 1893 will be omitted because of the Congress during the World's Fair), we think that at least half of the whole day's session of this Bureau should be given over to a thorough discussion of the best methods of *studying* and *teaching materia medica.* In order to elevate and dignify this important topic, and place it where it belongs in the very forefront of Homœopathy, we respectfully solicit answers from you, as we do from all teachers of our materia medica the world over, to *all* the following questions:

I. What advice do you give concerning materia medica to a student beginning medicine by a year's preliminary study?

II. Which is the best method of teaching materia medica—(*a*) for the preceptor to his student; (*b*) for the teacher to his classes in the college; (*c*) give an outline of your method of studying or teaching a drug in the class-room?

III. Which is the best place for teaching therapeutics—(1) Hospital, (2) Dispensary, (3) Clinic, (4) Class-room, or (5) Bedside, and how should it be done?

IV. Do you teach the potency of the remedy studied? If not, why not? If you do, how do you explain the potency you advocate?

V. When should *The Organon* be taught, and how?

This does not involve a long essay, unless you desire to contribute such to the Bureau over and above these answers. Please give this your prompt attention, in order that a complete *résumé* of how our therapeutics are taught may be carefully prepared.

Yours fraternally,

WM. E. LEONARD, M. D.,
*Secretary.*

